# Data of the maximum solid solubility limits of binary systems of elements

**DOI:** 10.1016/j.dib.2019.104515

**Published:** 2019-09-18

**Authors:** R. Goodall

**Affiliations:** Department of Materials Science & Engineering, The University of Sheffield, United Kingdom

**Keywords:** Periodic table of the elements, Alloy design, Solid solubility

## Abstract

This paper gives the available data for the maximum equilibrium solubility limits of each of the first 83 elements (H to Bi) of the periodic table in each of the others. This is expressed in the form of the maximum value of the equilibrium solid solubility for terminal solid solutions, expressed in atomic percentage (at%), occurring at any temperature where the room temperature phase of the pure element acting as the solvent is stable. The values thus represent the compatibility between different elements in the formation of alloys and similar combinations, and will be of use for research into fundamental solid state physics relating to elemental interactions, for the interpretation of phase structures in materials research and for the design of alloys and materials involving elemental mixing of the elements.

**Table:** Specifications Table

Subject area	*Chemistry*
More specific subject area	*Materials Science*
Type of data	*Table*
How data was acquired	*Survey and analysis of literature binary phase diagrams*
Data format	*Analyzed data, tabulated*
Experimental factors	*Each phase diagram was researched and assessed for detail and reliability*
Experimental features	*Where not already reported, diagrams were graphically analyzed for solubility data*
Data source location	*University of Sheffield, Sheffield, UK*
Data accessibility	*Raw data included in article, see Excel file in supplementary material*

**Table:** 

**Value of the Data** • Many phase diagrams showing the equilibrium structures formed when mixing pairs of elements have been assessed, yet, while these are available individually, collected data across all systems, such as presented here, are not accessible without time consuming search. • Solubility data for the solid phase allows interpretation and prediction of the behavior of elements combined into alloys. It is therefore of great use in physical metallurgy and alloy design, with application to the development of new materials including lightweight structural alloys, high temperature alloys, High Entropy Alloys, materials for hydrogen storage and novel functional materials such as permanent magnets, thermoelectric and magnetocaloric materials, as examples. • The collation of this data displays the underlying trends arising from the role of the electronic structure in alloying (as displayed in the periodic table), and analysis of the data may reveal further detail and exceptions to established trends, the study of which may lead to refinement of theory in solid state physics.

## 1 Data

The data presented in this article are for the maximum equilibrium solubility limits of each of the first 83 elements (H to Bi) in each of the others. These are taken to be the maximum value of solid solubility for the terminal solid solutions, expressed in atomic percentage (at%), occurring at any temperature where the room temperature phase of the pure element acting as the solvent is stable. The values thus represent the compatibility between different elements in the formation of alloys and similar combinations, with higher values indicating a greater compatibility. A value of 100 at% indicates complete solid solubility, while a value of 0 at% indicates immiscibility in the solid state. The data are presented in the form of a table, with each of the elements listed in order. Each row corresponds to a particular element acting as a solvent, and the columns show the solubility of each of the other elements as a solute in the system. Values have been given to the precision of the original measurements or predictive output on which the assessment is made. Data have been found for 3787 systems, and where no values are reported, the cell is left blank. The full data set is summarized below in 4 tables (see Table 1, Table 2, Table 3, Table 4) (split in this manner for legibility in the print version), each showing a quadrant of the overall matrix, while the complete table of the Raw data is included in Excel form for ease of data reuse in the Supplementary Material.

**Table 1:** Maximum equilibrium solubility limits in binary systems involving the first 42 elements (H to Mo) as both the solvent and the solute. Data are shown as the solubility limit, in atomic percent (at.%) of the element “B” in element “A”.

Element A	Element B																																									
	H	He	Li	Be	B	C	N	O	F	Ne	Na	Mg	Al	Si	P	S	Cl	Ar	K	Ca	Sc	Ti	V	Cr	Mn	Fe	Co	Ni	Cu	Zn	Ga	Ge	As	Se	Br	Kr	Rb	Sr	Y	Zr	Nb	Mo
H													0																													
He																																										
Li	0			0		0	0	0			0	76	0	0		0	0		0	2.2	0.1	0	0	0	0			0	0	0	0	0		0			0	0		0	0	0
Be	0		0		0	0		0			0	0	0	0					0	0		0	0	0.1		0.9	2.7	10	9.5	0	0	0			0		0	0	0	0	0	0
B				0		0	0					0	0	2.5		0				0	2	0	0	2	4	0	0	0	0	0	0	4	0					0	0	0	0.5	0
C			0	0	0						0		0	0						0	0	0	0	0	0	0	0	0	0			0	0	0			0	0	0	0	0	0
N						0		10																																		
O							0																																			
F																																										
Ne																																										
Na	0		0	0.1		0		0	0			0	0			0	0		1	0		0	0	0					0	0	0	0		0	0		0	0		0	0	0
Mg	11		18	0	0	0	0	0			0		12	0	0				0	0.8	29	0.1	0	0	1.1	0	0	0	0	2	4	0					0	0	3.4	0.7	0.1	0
Al	0		14	0	0	0	0	0			0	19		1.5	0	0			0	0	0.2	0.7	0	0	0.6	0	0	0	2.5	67	9	2.6	0	0				0	0	0	0	0
Si	0		0	0	2.6	0	0	0				0	0		2.5	0				0	1	0	0	0	0	0	0	0	0	0	0	100	3.5	0				0	0	0	0	0
P												0	0	0		0										0	0	0	0	0		0	35	0								0
S			0		0						0		0		0				0			0	0		0		0	0	0	0		0	0	12	0		0			0	0	0
Cl											0									0										0	0				100							
Ar																																										0
K	0		0		0	0		0	0		4.7	0	0			0	0			0		0	0	0					0	0	0	0	0	0	0		100	0		0	0	0
Ca	0		10	0	0	0	0	0	0		0	0	0	0			0		0		0	0	0		0.7	0		0	0	0	0	0			0		0	100	0			0
Sc			0		0	2.6		11				28	10	0		0	0			1.4		11	0.5	0	0	0.5	0	1	1.1		0	2.7			0			1.6	100	100	0	0.5
Ti	7.5		0	0	0	1.2	25	32			0	1.6	52	0.5	0	0			0	0	7.8		2	0.6	0.4	0	0.8	0	1.3	0	16	4.1		0.2			0	0	1	100	3	0.4
V	42		0	15	0	4.1	14	17			0	0	55	4.8		0			0	0	0.3	100		100	100	100	19	24	4.9	0	41	4.5		0			0	0	0	7.9	100	100
Cr	4		0	9.1	1	0	4.4	0			0	0	30	13	0	0	0		0		1.5	100	100		71	100	50	32	0	2	16	12	6	0			0		1	2.5	6.4	100
Mn			0		1	6.5	0.8	0				0	2	7.1	0	0				0	0	12	9	9		30	3	14	0.3	2	1	1.5	0	0				0	0	2		21
Fe	0		0	28	0	0.1	0.4	0			0	0	45	3.2	4.8	0			0	0	0.7	10	100	100	3		77	5.5	6.7	42	36	18	10	0			0	0	0.6	0.1	0.3	24
Co	0.5		26	12	0	4.5	0	0			0	0	17	19	0	0			0	8	0.6	14	2	44	20	2		35	20	2.8	1	18	2	0					0	0	0	0.9
Ni	0.9			24	0	2.9	0	0				0	21	16	0	0	0			0	1.6	15	43	50	100	100	100		100	39	24	16	5	0				0	0	1.8	13	27
Cu	0.2		22	14	0.3	0		0			0	2.7	19	11	4	0	0		0	0	0.5	10	0.4	0.6	100	4.6	7.5	100		38	21	12	6.8	0				0	0	0	0.9	0
Zn	0		0.9	0	0			0			0	0	2.9	0	0	0	0		0	0	0	0	0.2	0	1	0	0	0	2.8		2.4	0	0	0	0		0	0	0	0	0	0
Ga			0	0	0	0					0	0	0	0	0	0	0		0	0	0	0	0	0	0	0.5	0	0	0	0.8		0	0	0	0		0	0	0	0	0	0
Ge			0	0	0	0		0			0	0	1.1	100	0.2	0			0	0	0	2.3	0	0	0	0	1.5	0	0	0	1.1		0.2	0			0	0	0.3	0	0	0
As					0	0						0	0	0	19	0			0					0	0	0.1	0		0	0	0	12		0			0					0
Se			0		0	0					0		0	0	0	13			0			0	0	0	0	0	0	0	0	0	0	0	0		0		0	0			0	0
Br				0							0					0	100			0	0									0				0			0	0				0
Kr																																										
Rb	0		0	0		0		0	0		0	0				0	0		100	0		0	0	0						0	0	0	0	0	0			0		0	0	0
Sr	5		0	0	0	0		0			13	0	0	0			0		0	100	0	0	0		0	0		0	0	0.5	0	0		0	0		0		0			0
Y				0	0	7	3	15				20	0	0			0			0.5	100	0.9	0.3	0.7	0	1.6	0	0	0	0	0	1						6.4		1.8	0.1	0
Zr	5.2		0	0	1.5	0	23	31			0	0.4	8.3	0					0		100	100	2	1	0	0	5	0.2	0.1	3.2	0.8	1					0		0		0.7	0
Nb	49		0	13	1.7	5.2	17	9			0	0.2	20	3.5		0			0		0	100	100	25		9.1	5.3	5	5.6	0	19	11		0			0		0.1	100		100
Mo	0	0	0	1	0.9	1.1	1	1	0	0	0	0	20	3	16	3	0	0	0	0	0.5	100	100	100	37	31	7.1	2.9	0	0	15	5	0	0	0	0	0	0	0	9	100

**Table 2:** Maximum equilibrium solubility limits in binary systems involving the first 42 elements (H to Mo) as the solvent and elements 43 to 83 (Tc to Bi) as the solute. Data are shown as the solubility limit, in atomic percent (at.%) of the element “B” in element “A”.

Element A	Element B																																								
	Tc	Ru	Rh	Pd	Ag	Cd	In	Sn	Sb	Te	I	Xe	Cs	Ba	La	Ce	Pr	Nd	Pm	Sm	Eu	Gd	Tb	Dy	Ho	Er	Tm	Yb	Lu	Hf	Ta	W	Re	Os	Ir	Pt	Au	Hg	Tl	Pb	Bi
H																																									
He																																									
Li		0	0	0	9	18	0	0	0	0			0	0	0	0	0	0	0	0										0	0				0	0	0		0	0	0
Be		0		5	0		0	0	0	0			0	0														0		0	0	0	0				0.3				0
B	0	0	0	0		0	0	0	0					0	0	0	0	0	0	0	0	0	0	0	0	0	0	0	0	1	0.4	1	0	0		0	0	0	0	0	0
C		0	0	0				0	0				0	0	0	0	0	0			0	0	0	0	0	0		0	0	0	0	0			0	0				0	0
N																														0											
O																																									
F																																									
Ne																																									
Na				0	0	0	0	0	0	0	0		0	5.7																0	0					0	0	0	1.1	0	0
Mg				0.2	3.8	100	19	3.4	0	0			0	0	0	0.1	0	0.8		0	0	4.5		4.8	5.4	6.9	6.3	1.2	8.8	0					0.2	0	0.1	1.2	16	7.7	1
Al		0.5	0	0	23	0	0	0	0	0	0		0	0	0	0	0	0	0	0	0	0	0	0	0	0	0	0	0	0	0	0	0		0	0	0.1	0	0	0	0
Si		0	0	0	0	0	0	0.1	0	0				0		0	0	0		0		0	0	0	0	0	0	0	0	0	0	0	0		0	0	0		0	0	0
P			0	0	0			0									0																	0	0	0	0				0
S		0			0	0	0	0	0	0.3	0		0				0			0	0			0				0				0				0	0	0	0	0	0
Cl					0		0				0		0	0																								0			0
Ar																																									
K					0	0	0	0	0	0	0		100	0																0	0						0	0	0	0	0
Ca				0	0	0	0	0	0		0		0	100	0	0		0.1		0	55	0	0.5	0		0	0.4	100	0.3							0		0	0	0	0
Sc		1	2	0	0		15	4.5		0	0			2	15	12		100			0	100	100		100	100				100	0.1	0	0		1	0	0			0	
Ti	1	0.1	0.1	1	4.7		10	11	12	0.9			0	0	1	1.2		1			0	6	1			0.1	0.1	0		100	3.6	0.2	8	20	15	2	1.7	0		4	0.5
V	44	25	18	38		0	5.5	16		0			0	0	0	0.6	0.1	0	0.1	0.1	0	0	0.1	0.1	0.1	0	0.1	0	0	4	100	100	65	37	22	11	33	0	3.4		
Cr		32	20	1		0	0	2	5	0	0		0		2.5		0	1.5	0	0	0	0	1	0	3.7	0	0	0	0	2	4.3	100	50	30	11	6.8	3	0		0	0
Mn			1	3.7	0			2	1.5	0				0	0	0	0	0	0	0	0	0	0	0	0	0	0	1	0	0.2	2	10	5.5		5	1	0		0	0	0
Fe		4	19	3.5	0	0	0.3	9.2	5	1.5			0	0	0	0	0	0	0	0	0	1	0	0	0	0	0	0	0	0.7	0.8	15	8	3	8		1	0		0	0
Co		100	100	20	0	0.3	0	0	0	0					0	0	0	0		0		0	0	0	0	0		0	0	0.5	4	0	100	100	100	100		0		0	0
Ni	29	31	100	100	1.4	0	8	11	10	0				0	0	0	0	0		0	0.8	0		0	0	0		0		1	11	18	17	14	100	100	100	0	2.5	0.1	
Cu	0	0	100	100	4.9	2.2	11	9.1	5.8	0	0		0	0	0	0	0	0	0	0	0	0	0	0	0	0	0	0	0	0.3	0	0	0		8	100	100	5	0.3	0	0
Zn	0	0			4	1.5	0.1	0	0	0	0			0	0	0	0	0		0	0	0	0	0	0	0	0	0	0							0	5.5	3	0	0	0.1
Ga				0	0	0	0	0	0	0	0		0	0	0	0	0	0	0	0	0	0	0	0	0	0	0	0	0	0			0			0	0	0	0	0	0
Ge		0	0	0	0	0	0	1.1	0	0	0		0	0	2		0	1		0	0	0	0	0	0	0	0	0	0	0		0	0			0	0	0	0	0	0
As				0	0	0	0	15	100	0								0		0	0	0	0	0	0	0	0	0	0		0			0		0	0		0	0	0
Se		0	0	0	0	0	0	0	0	100	0		0	0	0	0	0									0	0	0			0			0			0	0	0	0	0
Br					0		0	0		0	0				0	0	0														0						0	0		0	0
Kr																																									
Rb					0	0	0	0	0	0	0		100	0																0	0						0	0	0	0	0
Sr					0	0	0	0	0	0	0		0	22				0.3																		0	0	0	0	0	0
Y		0		0	0		8	1.7	1.7		0			7.9	100			100		100		100	100	100	100	100		0	100	1	0		0	0	0	0				2	0
Zr		0.6	0	1	1.3	18	10	6.5	0.8	0			0		0	2.8		3.4			0	3	4.1	7	3.7	28		0	4.9	100	0.7	4	1.5	1.5	1	1.5	2	0		5	4
Nb		40	21	36			0	11	20	0	0		0		0.1	0				0.1	0					0		0	0	100	100	100	46	19	12	12	36	0	0		
Mo	49	33	20	6	0	0	0.1	0	0	0	0	0	0	0	0.1	0.3	0.3	0.3	0.3	0	0	1	0.7	0.6	0.6	0.5	0.5	0	4	23	100	100	38	18	16	15	0.4	0	0	0	0

**Table 3:** Maximum equilibrium solubility limits in binary systems involving elements 43 to 83 (Tc to Bi) as the solvent and the first 42 elements (H to Mo) as the solute. Data are shown as the solubility limit, in atomic percent (at.%) of the element “B” in element “A”.

Element A	Element B																																									
	H	He	Li	Be	B	C	N	O	F	Ne	Na	Mg	Al	Si	P	S	Cl	Ar	K	Ca	Sc	Ti	V	Cr	Mn	Fe	Co	Ni	Cu	Zn	Ga	Ge	As	Se	Br	Kr	Rb	Sr	Y	Zr	Nb	Mo
Tc					0	7.2																14	8					46	0	0												25
Ru			0	1	1.8	3							4	4.5	0	0					1.5	14	30	52	51	77	100	49	0	0	0			0					0	2	29	52
Rh			0		0	1.5							0	2.3	0	0					11	14	20	32		100	100	100	100			0		0						8	17	15
Pd	0.1		4.4	1.1	18	8					2	25	19	0	0	0	0			0	15	15	58	50	28	100	100	100	100	20	11	3	0	1.5					13	14	30	46
Ag		0	61	0.4	0	0		0			0	24	20	0	0	0.1	0	0	0	0	10	4	1	0	55	0	0.8	0.3	14	40	19	9.6	8.8	0	0	0	0	0	1.6	5.3		0.2
Cd			30		0			0			0	100	0	0	0	0	0		0	0	0	0	0	0	0	0		0	0.2	4.4	0	0	0	0			0	0	0	0		0
In			1.6	0	0						2.9	5	0	0	0	0	0		0	0	0	0	0		0	0	0	0	0	2	2.3	0	0	0	0		0	0	0	0	0	0
Sn			0	0	0	0		0	0		0	0	0	0	0	0	0		0	0.4	0	0	0	0	0	0	0	0	0	0	6.5	0	0	0	0		0	0	0	0	0	0
Sb			0	0	0	0		0			0	0	0	0	0.1	0			0	0		0		0	0	0	0	0	0	0	0	6.2	100	0			0	0	0	0	0	0
Te			0	0				0			0	0	0	0		14	0		0		0	0	0	0	0	0	0	0	0	0	0	0	0	100	0		0	0	0	0	0	0
I											0		0			0	0		0	0	0			0					0	0	0	0		0	0		0	0	0		0	0
Xe																																										
Cs	0		0	0		0		0	0		0	0				0	0		100	0		0	0	0					0		0	0		0			100	0		0	0	0
Ba	58		0	0	0	0		0			1.2	0	0	0	0		0		0	100	0	0	0		1.2	0		0	0	0	0	0		0	0		0	100	0.2			0
La	1.4		0		0	4.5						1.1	0			0	0			0	11	0	1.4		0	0	0	0	0	0	0	0		0	0				100	0	0	0
Ce			0		0	4.5	3					8.1	0	0		0				0.3	17	0	0		5.2	0	0	0	0	0	0	0		0	0							0
Pr			0		0	4.5		0				9.2	3	0	0.3	0	0					3	0	0		0	0	0	0	1.5	4	0		0	0							0
Nd	12		0		0	0		0				8.9	2.1	0						3.5	100	0	0	3	2.5	0	0	0	0	1		0.5	1					5.8	100	1.5		0
Pm			0		0								0										0.2	0	0	0			0		0											0
Sm			0		0				0			3	0	0		0				0.2			0	0	0	0.5	0	0	0	0	0	0	0						100		0.1	0
Eu					0	0		0				0	0			0				100	0	0	0	0	0	0		0	0	0	0	0	0							0	0	0
Gd					0	0						14	0.5	0			0			0.2	100	0.3	0	0	0	0	0	0	0		0	0		0					100	3.4		0
Tb					0	0		0				12	0	0						9	100		0.2	3	0	0	0		0		0	0	1						100			0
Dy					0	0						13	0	0		0	0			0.2			0.3	0	0	0	0	0	0		0	0	1						100	1.5		0.6
Ho					0	0						15	0	0			0				100		0.5	2	0	0	0	0	0	0	0	0	1						100			0.6
Er	40				0	0		0				6.9	0	0			0			0.2	100	3	0	0	0	0	0	0	0	0	0	0	1	0					100	42	1	0.5
Tm					0							13	0	0		0	0			6.4		1	0.4	0	0	0			0	0	0	0	1	0								0.7
Yb					0	0			0			0	0.6	0		0	0			100		0	0	0	0	0	0	0	0	0	0	0	0	0					0	0	0	0
Lu					0	0						12	0	0						6.4			0.5	0	0	0	0		0	0	0	0	1						100		0.5	4
Hf	9		0	0	1.5	14	29	25			0	0	27	0					0		100	100	3.4	1.7	2		3	0.9	0.3								0		4	100	5.2	2
Ta	34		0	0	2	7	11	5.7			0		6.9	10								100	100	27		8.5	21	21	0				0	0	0		0		0	100	100	100
W				5.8	0	1	0	0					15	3		0						100	100	100	12	4	3	0.3	0			2.5							0	3.6	100	100
Re				10	0	11							2.8	9.2							6	3	13	23		0	100	17	0		0	0							0	4	4	13
Os					0	2									0							21	48	65		75	100	12					0	0					0	8	27	52
Ir			0			3.1							20	0	0						11	11	19	27		100	100	100	6.4										0	9	15	23
Pt				0	0	2.5					1		16	0.7	0	0				0		11	57	69	38	100	100	100	100	22	14	6.7	0					0	0	13	20	44
Au	0		17	0.2	5	0.1	0				0	0	13	0	0	0	0		0	0	9.4	11	61	47	31	74	23	100	100	35	12	3	0	0	0		0	0	2	8.1	57	1.3
Hg			0		0	0					0	0	0			0	0		0	0		0	0	0	0	0	0	0	0	0.3	0	0		0	0		0	0	0	0.3	0	0
Tl			1.4		0						27	2.1	0	0	0	0	0		4	2			0		0			0	0	0	0	0	0	0				4.5	0		0	0
Pb			2.9		0	0		0			20	5.7	2.3	0		0	0		0	0	0	0		0	0	0	0	0	0	0	0	0	0	0	0		0	0	0	0		0
Bi			0	0	0	0		0			0	0	0	0	0	0	0		0	0	0	0	0	0	0	0	0	0	0	1.6	0	0	0.6	0	0		0	0	0	0	0	0

**Table 4:** Maximum equilibrium solubility limits in binary systems involving elements 43 to 83 (Tc to Bi) as both the solvent and the solute. Data are shown as the solubility limit, in atomic percent (at.%) of the element “B” in element “A”.

Element A	Element B																																								
	Tc	Ru	Rh	Pd	Ag	Cd	In	Sn	Sb	Te	I	Xe	Cs	Ba	La	Ce	Pr	Nd	Pm	Sm	Eu	Gd	Tb	Dy	Ho	Er	Tm	Yb	Lu	Hf	Ta	W	Re	Os	Ir	Pt	Au	Hg	Tl	Pb	Bi
Tc										0																						12	100								
Ru			60	8				0	0	0			2		1.5	2		0		0		0	0	0		0		0	2	5	28	48	100	100	49	21	0	0			
Rh		35		100	10			15	6.5	1					0	0		0	0	0	0	0		0		0		0	1	5	18	19	12	55	100	100	0.3	0		0	
Pd		21	100		100	26	19	17	17	16				0		13	9	3		10		12	12	12	12	13		13	13	23	18	22	16	2.9	100	100	100	14		17	
Ag		0	0	100		42	21	12	7.2	0		0		0	0	0.1	0.1	0.3	0	1	0	0.7	1.1	1.3	1.4	3.1	4.6	1.9	5.8			0	0	0	0	40	100	37	7.8	2.8	2.6
Cd				0	7		1.4	0.2	0	0	0		0	0	0	0	0	0	0	0	0	0						0								0	4	25	0	0	0
In			0	0	0	6		12	0	0	0		0	0	0	0	0	0	0	0	0	0	0	0	0	0	0	0	0		0				0	0	0	4.5	31		8.2
Sn		0	0	0	0	1.1	0		0	0	0		0	0	0	0	0	0		0	0	0	0	0	0	0	0	0	0	0	0		0			0	0	1	0	0	13
Sb		0	0	0	0	0	0	12		1.3			0		0	0	0	0		0		0	0	0	0	0	0	0.9	0			0	0		0	0	0	0	0	2	100
Te	0	0	0	0	0	0	0	0	0		0		0	0	0	0	0	0		0	0	0		0	0	0	0	0		0	0	0	0	0	0	0	0	0	0	0	0
I						0	0	0		0			0	0								0	0		0	0												0	0		0
Xe																																									
Cs						0	0	0	0	0	0			0			0													0	0						0	0	0	0	0
Ba				0	0	0	0			0	0		0		0.2		0	0	0	0	100							100								0		0	0	0	
La		0	0		0	0	0	0	0	0				0		100	100	100	100	100		100		21				0			0	0	0		0	0	0	0	0	0	0
Ce		0	0	0	0		0	0	0	0	0				100		70	100	100	2	4.4	3								0	0	0			0	0	0	0	0	0	0
Pr				0	1.3		5.6	3.5	2	0			0	0	100	100		100	100			100		100						0	0	0	0	0.2	0	0.1	1	0.9	2.5	2.2	0
Nd		0	0		0.9	0	5	2	0	0				0	100	100	100		100	100		100		100		100		2.2			0	0			0	1	0		3	2.5	0
Pm							5							0	100	100	100	100				100		100													0		4		
Sm		0	0	0.7	0	0	0	0	0	0				0	7	20		13				40	19	15	13		9		7		0	0			0	0	0.2			0	
Eu			0	0	0	0	0	0		0					5	0									0			100		0	0	0				0	0			0	
Gd		0	0	3.7	0	5	7	0	4	0	0				100	30	100	100	100	100			100	100	100	100	100	19	100		0	0	0			0	0		7.2	1	0
Tb				0	0		9	0	0		0									100		100		100	100	100	100		100		0	0	1		0	0	0	0		0	1
Dy		0	0	0	0		8	3	0	0	0				62		100	100	100	100		100	100		100	100	100		100		0	0				0	0		5	3.1	0
Ho				0	0		8	2.5	0	0	0									100	0	100	100	100		100	100		100		0	0			0	0			5		1
Er		0	2	0	0		7	1.4	1.4	0	0							100				100	100	100	100		100		100	2.5	0	0	0		0	0	0	0	6		1
Tm				0	0		10	0	2.5	0										100		100	100	100	100	100			100		0	0			0	0	0		0		0
Yb		0	0	0	0	1.4	0	0	0	0				10	0			1.7				1.4			0				0	0	0	0		0	0	0	0	0	6	0	1
Lu		2		0	0			0	3											100		100	100		100	100	100	15			0	0			0	0				0.8	
Hf		2	3	1	0			14	0	0			0			0.5	0							100		7.5		0			5.1	0	0.8	1	1.4		5	0			
Ta		49	16	25			0	9		0			0								0		0				0	0	0	100		100	47	78	7.3	9	20		0		0
W	55	23	6	5					0	0					0	0	0	0		0	0	0	0	0		0	0	0	0	7.5	100		37	19	10	4.7	0				0
Re	100	100	24					0	0	0					0							0	1			0				11	4	20		100	44	44	0	0			0
Os		100	30	5.2	0					0							0											0		13	19	54	100		38			0			0
Ir		45	100	100	0		0		0	0					0	0	0	0	0	0		0	0	0	0	0	0	0	0	10	16	19	36	44		100	2	0			0
Pt		74	100	100	13		13	11	11	3				0	0	0		1		0	0	0.7	0	0	0	0	0	1	0		19	63	48	25	100		100		0	0	0
Au	0	0	1.6	100	100	33	14	7.3	0	0.2			0		0	0	0.1	0	0	0.3	0	0.7	1.5	2.3	3.9	5.7	0	7.1	7.7	5	35	0	0	0	0.1	100		20	1	0.1	0.1
Hg		0	0	0	0	3.5	19	0	0	0	0		0	0	0		0	0		0		0	0	0	0	0		0		0			0	0	0	0	0		0	0	0
Tl				0	0	4	13	3	3	0	0		0	1.7	0	0	0	0	0	0		0	0	0	0	0	0	0	0		0					0	0	2.5		4.3	1
Pb			0	0	0.2	5.9	71	28	7	0			0	0	0.1	0	0	0		2	0	0	0	0				0	0			0				0	0.2	25	90		25
Bi		0	0	0	0	0	0	3	100	0	0		0	0	0	0	0	0		0		0	0	0	0	0	0	0	0.1	0	0	0	0	0	0	0	0	0	0	0	

## 2 Experimental design, materials, and methods

The data presented have been obtained by an analysis of binary phase diagrams, obtained by a thorough survey of the literature. The majority of the diagrams used have been taken from major reference works on the subject [1], [2], [3], with the most recent report in each case taking precedence. Additional reports of mutually insoluble pairs and some more up to date data have been taken from the wider literature, with the full sources identified with the data (accessible with the Excel file containing the complete data). In each case, the reported data have been assessed to ensure a good standard of confidence is maintained, allowing phase diagrams derived from experimental investigation and those generated by thermodynamic calculation, but ensuring in either case that the reports are sufficiently detailed and supported by reference to wider literature. In some cases, maximum solubility limits are reported explicitly, and these are then taken to the accuracy at which they are reported. In other cases, where the diagrams only are shown they are graphically analyzed to determine the relevant values, which are given to the precision of other values provided in that study, or the dimensions of the relevant line, whichever is the lesser. All values are given in atomic percent, and where originally given in weight percent they are converted using the standard atomic masses of the elements concerned. Where no data have been found, the corresponding cells have been left blank.
